# Contouring the accessory parotid gland and major parotid glands as a single organ at risk during nasopharyngeal carcinoma radiotherapy

**DOI:** 10.3389/fonc.2022.958961

**Published:** 2022-11-11

**Authors:** Xin-Ling Cai, Jiang Hu, Jun-Tian Shi, Jin-Shu Chen, Shou-Min Bai, Yi-Min Liu, Xiao-Li Yu

**Affiliations:** ^1^ Department of Radiation Oncology, Shenshan Medical Center, Sun Yat-Sen Memorial Hospital, Sun Yat-Sen University, Shanwei, China; ^2^ Department of Radiation Oncology, Sun Yat-Sen University Cancer Center and State Key Laboratory of Oncology in Southern China, Collaborative Innovation Center of Cancer Medicine, Guangzhou, China; ^3^ Guangdong Provincial Key Laboratory of Malignant Tumor Epigenetics and Gene Regulation, Sun Yat-sen Memorial Hospital, Sun Yat-sen University, Guangzhou, China; ^4^ Department of Radiation Oncology, Sun Yat-sen Memorial Hospital, Sun Yat-sen University, Guangzhou, China

**Keywords:** nasopharyngeal carcinoma, accessory parotid gland, dosimetry analysis, contouring, xerostomia

## Abstract

**Background and purpose:**

No research currently exists on the role of the accessory parotid gland (APG) in nasopharyngeal carcinoma (NPC). We thereby aimed to assess the effects of APG on the dosimetry of the parotid glands (PGs) during NPC radiotherapy and evaluate its predictive value for late xerostomia.

**Material and methods:**

The clinical data of 32 NPC patients with radiological evidence of the APG treated at Sun Yat-sen Memorial Hospital between November 2020 and February 2021 were retrospectively reviewed. Clinically approved treatment plans consisted of only the PGs as an organ at risk (OAR) (Plan1), while Plan2 was designed by considering the APG as a single organ at risk (OAR). The APG on Plan1 was delineated, and dose–volume parameters of the PGs alone (PG-only) and of the combined structure (PG+APG) were analyzed in both plans. The association of such dosimetric parameters in Plan1 with xerostomia at 6–9 months post-radiotherapy was further explored.

**Results:**

Fifty APGs were found, with a mean volume of 3.3 ± 0.2 ml. Significant differences were found in all dosimetric parameters between Plan1 and Plan2. The mean dose and percentage of OAR volumes receiving more than 30 Gy significantly reduced in Plan1 itself (PG-only vs. PG+APG, 39.55 ± 0.83 Gy vs. 37.71 ± 0.75 Gy, and 62.00 ± 2.00% vs. 57.41 ± 1.56%, respectively; p < 001) and reduced further in Plan2 (PG+APG, 36.40 ± 0.74 Gy, and 55.54 ± 1.61%, respectively; p < 0.001). Three additional patients met the dose constraint in Plan1, which increased to seven in Plan2. With APG included, the predictive power of the dosimetric parameters for xerostomia tended to improve, although no significant differences were observed.

**Conclusion:**

APG is anatomically similar to the PGs. Our findings suggest the potential benefits of treating the APG and PGs as a single OAR during radiotherapy (RT) of NPC by improving PG sparing.

## Introduction

Nasopharyngeal carcinoma (NPC) is a radiosensitive cancer characterized by its unique geographic distribution, with particularly high incidences in Southern China (1, 2). While radiotherapy (RT) represents the mainstay treatment for non-metastatic NPCs, radiation-induced xerostomia is a common long-term complication that can greatly affect the quality of life of patients (3). Despite the advent of more advanced RT techniques such as intensity-modulated RT (IMRT), the incidence of grade III–IV xerostomia remained between 13.9% and 27.5% among patients with mild-to-severe skull-base invasion (4). This is mainly attributed to radiation damage of the salivary glands, particularly the parotid glands (PGs). Accurate delineation of the PGs is thus the cornerstone for their protection during RT.

Increasing attention has been paid to the protection of the PGs during RT. Several studies have developed the split-parotid delineation approach to spare specific regions of the organ, including the stem and progenitor cells and the superficial lobe (5–7). However, the accessory parotid gland (APG), which has been found as a fairly common anatomical variant with a prevalence of 21%–56% (8, 9), is rarely mentioned in the literature. Based on cadaveric studies, no appreciable histopathological differences from the PGs have been reported (8), and both serous and mucous acini have been identified, suggesting that APG may have similar functions as PGs (9). In most cases, the APG drains into Stensen’s duct (parotid duct) through an accessory duct (10). Nonetheless, current guidelines for the delineation of organs at risk (OAR) (11, 12) do not account for the APGs, and whether they should be included in the target volume of PGs remains unknown.

A strict dose constraint is essential to minimize the radiation exposure of the PGs. Recent guidelines have recommended a mean dose (Dmean) of ≤26 Gy, with maximum acceptance criteria of <30 Gy for ≥50% (D50≤30Gy) of at least one gland (12). However, with large tumors and gross nodal involvement, compromise of the PGs is often required to ensure adequate dose delivery to the target area. In addition, we observed clinical inconsistencies between xerostomia and dosimetric parameters of the PGs in patients with APGs. As such, the effects of considering APG as a homologous organ of the PGs on the dosimetry of the PGs, and subsequently the development of xerostomia, represent a question that needs to be addressed.

Our study thereby aimed to compare the dosimetric parameters of the PGs based on the inclusion of the APG during RT planning and evaluate its influence on late xerostomia development among NPC patients.

## Materials and methods

### Patients

The clinical and radiological data of biopsy-proven NPC patients treated at Sun Yat-sen Memorial Hospital between November 2020 and February 2021 were retrospectively collected. The inclusion criteria were as follows: 1) NPC stage I–IVa according to the 8th edition of the American Joint Committee on Cancer (AJCC8), 2) radiological evidence of the APG, 3) definitive treatment with IMRT, and 4) completion of treatment. Exclusion criteria were as follows: 1) lost to medical records and 2) incompletion of treatment. This study was approved by the local ethics committee of the institute.

### Target delineation and dose prescription

Contrast-enhanced CT imaging (SOMATOM Definition, Siemens Healthcare, Forchheim, Germany) was performed for IMRT planning. All patients were immobilized in the supine position with a head–neck–shoulder thermoplastic mask and a vacuum bag. The scans ranged from the superior margin of the frontal sinus to 2 cm below the clavicle with a slice thickness of 3 mm. Delineation of target volumes and OARs was performed based on recent international guidelines (11–13). The gross tumor volume included the primary tumor volume and any enlarged regional lymph nodes confirmed on CT and magnetic resonance imaging. The high-risk clinical target volume was defined as the gross tumor volume plus a 5–10-mm margin and the entire nasopharyngeal mucosa. The low-risk clinical target volume was defined as the high-risk clinical target volume plus a 5–10-mm margin and encompassed low-risk sites of microscopic extension such as the skull base, clivus, sphenoid sinus, parapharyngeal space, pterygoid fossae, posterior nasal cavity, pterygopalatine fossae, retropharyngeal nodal regions, and the elective neck area from level IB to V. A 3-mm margin was used to generate the corresponding planning target volume (PTV) and planning OAR volume (PRV).

IMRT was administered in 33 fractions, five fractions per week. The radiation doses to the gross tumor volume and the high- and low-risk clinical target volumes were 70, 60, and 54 Gy, respectively (PTV70Gy, PTV60Gy, and PTV54Gy, respectively). The dosimetric objectives of the PGs were set as either V30 ≤ 50% for at least one PG, or Dmean ≤ 26 Gy. The dosimetric parameters of other OARs were determined according to Radiation Therapy Oncology Group (RTOG) protocols 0225 and 0615 (14, 15).

### APG delineation and dosimetric data collection

**Figure 1:**
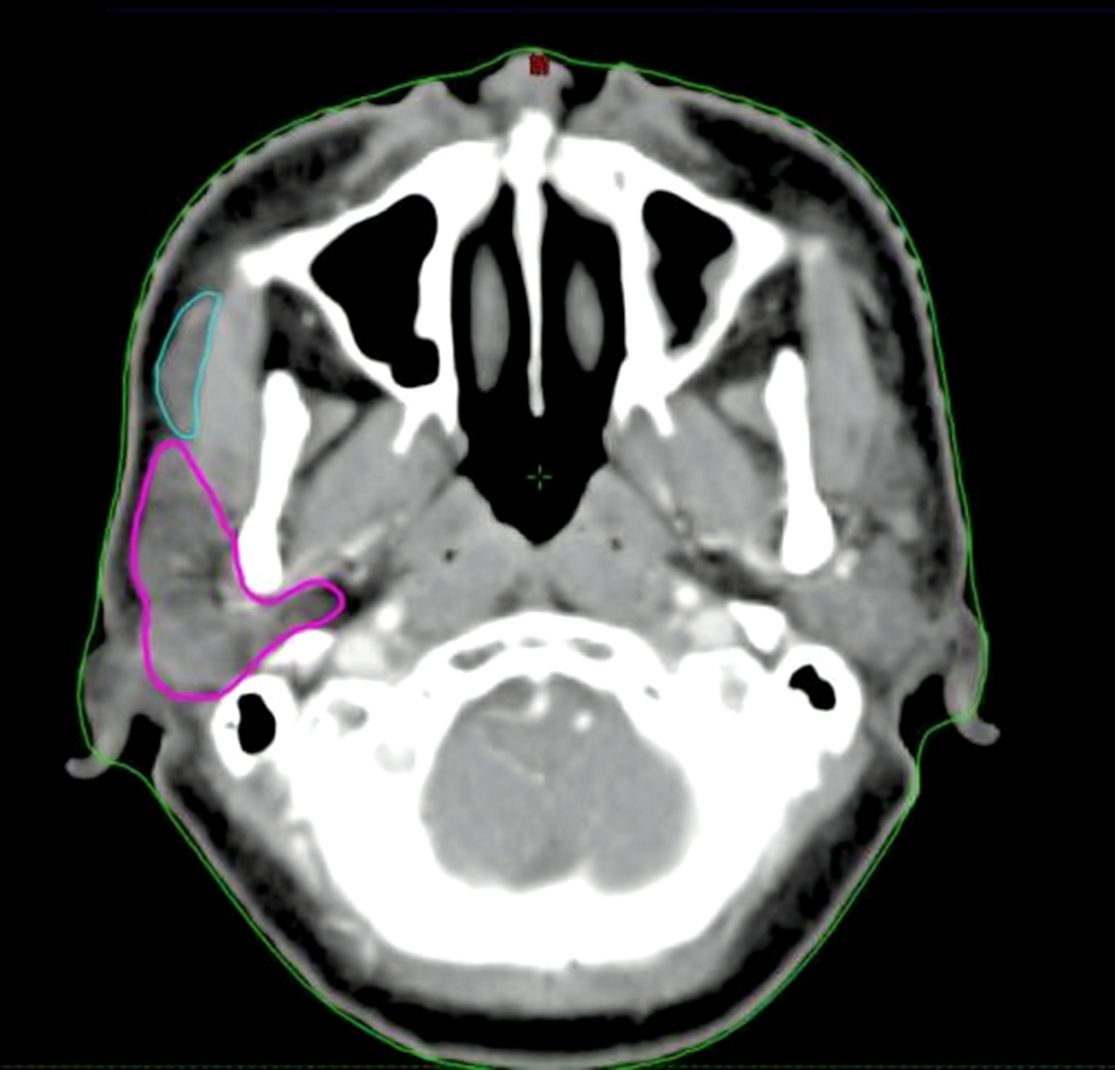
Delineation of the parotid glands (purple) and accessory parotid gland (cyan).

At this stage of analysis, two treatment plans were involved—Plan1 and Plan2. Plan1 represented the clinically approved treatment plans obtained from the Varian Trilogy system (Eclipse, version 13.5; Varian Medical Systems, Palo Alto, CA), whereby only the PGs were contoured (and protected) as an OAR. The APG was then outlined (without any attempts made to protect the gland) (**Figure 1**) to allow for dosimetric evaluation of the APG and the PGs and APG combined (PG+APG). Delineation of the APG was performed by two clinicians with >10 years of RT experience in NPC, and any disagreements were discussed and resolved by consensus. Plan2 was subsequently designed by intentionally treating the APG as an OAR. The target and OAR dose criteria from Plan1 were retained. All patients were treated using Plan1, while Plan2 was created for comparative purposes.

The dosimetric data of PG-only and PG+APG of both plans were subsequently compared. Dosimetric parameters were retrieved from dose–volume histograms and included the following: Dmean; the dose to 50% of the OAR volume (D50); percentage of the OAR volume receiving more than 26, 30, 33, and 45 Gy (V26, V30, V33, and V45, respectively); and the absolute OAR volume receiving lower than 20 Gy (V20cc).

### Xerostomia evaluation

Xerostomia was graded according to the RTOG late toxicity scale (16). The presence of xerostomia was evaluated at 6–12 months post-RT, and its association with all relevant dosimetric parameters in each of the two delineation approaches was compared. Clinically significant xerostomia was defined as those grades ≥2.

### Statistical analysis

All dosimetric parameters were compared using either the paired-samples T-test, Wilcoxon rank-sum test, or McNemar’s test, while clinical characteristics and xerostomia rate were compared using Fisher’s exact test. The predictive value of dosimetric parameters for xerostomia was assessed using the receiver operating characteristic (ROC) analysis, and areas under the ROC curve (AUCs) were compared using Delong’s test.

All statistical analyses were performed using SPSS Statistics version 23.0 and MedCalc version 12.0. Two-tailed p-values < 0.05 were considered statistically significant.

## Results

### Patient characteristics

Among a total of 136 NPC patients treated with IMRT at our hospital, 32 were identified to have the APG (23.53%) and were included in our study. A total of 50 APGs were found and were unilateral and bilateral in 14 and 18 patients, respectively. The mean age was 51 years (range, 29–71 years). The mean maximum diameter of LNs ipsilateral to the APG was 2.52 ± 0.25 cm. Locally advanced NPCs (stage III–IVa) were demonstrated in approximately 85% of the patients.

All baseline and clinical characteristics of the included patients are summarized in **Table 1**.

**Table 1:** Clinicopathological characteristics.

Variable	N = 32	
Sex	n	(%)
Male	23	71.88%
Female	9	28.13%
Age		
≥56	13	40.63%
<56	19	59.38%
Maximum diameter of unilateral LN*		
>2.5 cm	22	44.00%
≤2.5 cm	28	56.00%
T stage,		
T1	4	12.50%
T2	8	25.00%
T3	13	40.63%
T4	7	21.88%
N stage		
N0	3	9.38%
N1	8	25.00%
N2	18	56.25%
N3	3	9.38%
Clinical stage		
I	2	6.25%
II	3	9.38%
III	19	59.38%
IVa–b	8	25.00%
Clinical levels of LN*		
No	1	2.00%
II	11	22.00%
II–III	31	62.00%
II, IV	1	2.00%
II –IV	6	12.00%
Treatment, n (%)		
InC+CCRT	30	93.75%
CCRT	2	6.25%

LN, lymph node; CCRT, concurrent chemoradiotherapy; InC+CCRT, induction chemotherapy followed by concurrent chemoradiotherapy.

*Lymph nodes ipsilateral to the accessory parotid gland, N = 50.

### PG, APG, and target-overlapping volumes

The mean volumes were as follows: APG, 3.3 ± 0.2 ml (range, 1.3–8.6 ml); PG, 29.4 ± 1.3 ml (range, 15.8–47.7 ml); and PG+APG, 32.9 ± 1.4 ml (range, 19.0–56.0 ml). No overlaps between the APG and the target volumes were observed. In contrast, target-overlapping PG volumes were 0.60(0.20–1.00) ml, 0.55(0.20–1.00) ml, and 7.1 (6.0–9.1) ml for PTV70Gy, PTV60Gy, and PTV54Gy, respectively.

### Comparison of dosimetric parameters based on APG involvement

**Figure 2:**
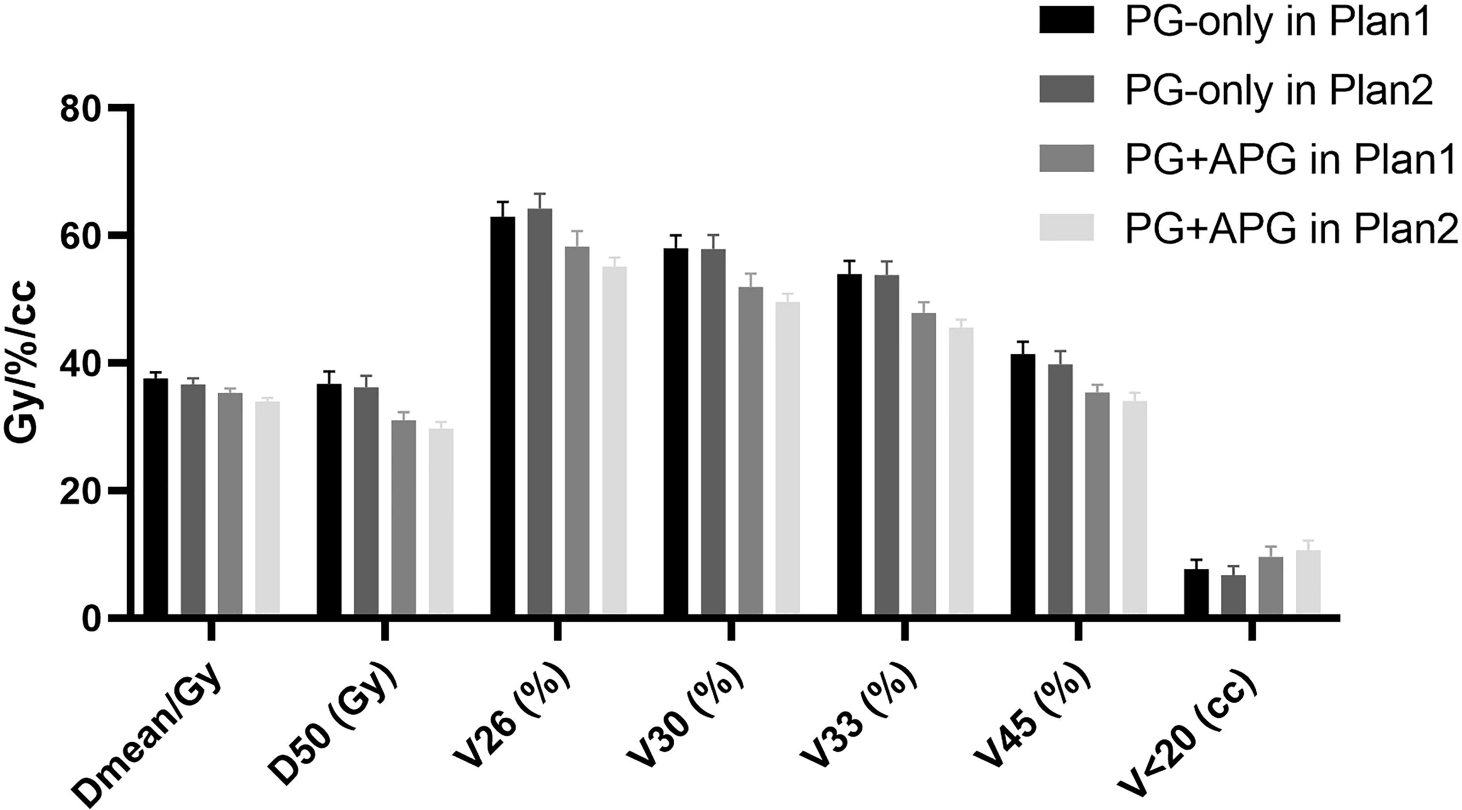
Dosimetric parameters in Plan1 and Plan2.

All dosimetric parameters between Plan1 and Plan2 are summarized in **Table 2**. A significantly higher Dmean of APG was observed in Plan1 compared to Plan2 (24.79 ± 0.85 Gy vs. 14.22 ± 0.41 Gy; p < 0.001). Both the Dmean and D50 of PG-only were significantly higher than that of PG+APG in Plan1 (39.55 ± 0.83 Gy vs. 37.71 ± 0.75 Gy; 39.31 ± 1.21 Gy vs. 35.37 ± 1.15 Gy, respectively; p < 0.001). Significant improvement was observed in all dosimetric parameters between PG-only in Plan1 and PG+APG in both Plan1 and Plan2 (**Table 2** and **Figure 2**). Significant improvement was also observed in the corresponding dosimetric parameters of PG+APG in Plan2 compared to Plan1 (p < 0.001). In Plan2, all the dosimetric parameters of PG-only, except for V26, were lower than those in Plan1 (**Figure 2**).

**Table 2:** Comparison of dosimetric parameters of PG-only and PG+APG in Plan1 and Plan2.

Variable	Plan 1			Plan2	
	PG-only	PG+APG	p-value	PG+APG	p’-value
Dmean (Gy)					
Mean	39.55 ± 0.83	37.71 ± 0.75	<0.001	36.40 ± 0.74	<0.001
Range	29.49–65.61	27.66–60.52		26.77–57.52	
D50 (Gy)					
Mean	39.31 ± 1.21	35.37 ± 1.15	<0.001*	34.31 ± 1.14	<0.001
Range	23.89–68.71	20.44–67.50		20.07–64.94	
V26 (%)					
Mean	68.07 ± 1.64	64.39 ± 1.62	<0.001	61.78 ± 1.55	<0.001
Range	47.00–99.84	41.74–94.57		41.40–94.20	
V30 (%)					
Mean	62.00 ± 2.00	57.41 ± 1.56	<0.001*	55.54 ± 1.61	<0.001
Range	42–99	36.99–89.76		36.80–88.30	
V33 (%)					
Mean	57.91 ± 1.68	52.80 ± 1.52	<0.001*	51.29 ± 1.62	<0.001
Range	38.89–98.81	34.22–87.63		33.50–85.00	
V45 (%)					
Mean	44.16 ± 1.77	39.18 ± 1.54	<0.001	37.25 ± 1.53	<0.001
Range	25.92–95.80	23.31–82.85		22.20–82.10	
V<20 (cc)					
Mean	6.65 ± 0.61	7.99 ± 0.73	<0.001	9.16 ± 0.76	<0.001
Range	0–19.99	0.05–25.27		1.20–25.10	
Organ Volume (cc)					
Mean	29.36 ± 1.28	32.85 ± 1.37	<0.001*	–	
Range	15.80–47.70	19.00–56.00		–	

PG-only, the parotid glands as an organ at risk; PG+APG, the parotid glands and accessory parotid gland as a single organ at risk; Dmean, mean dose; D50, dose of 50% OAR volume; V26, percentage of the OAR volume that received ≥ 26 Gy; V30, percentage of the OAR volume that received ≥ 30 Gy; V33, percentage of the OAR volume that received ≥ 33 Gy; V45, percentage of the OAR volume that received ≥ 45 Gy; V20cc, the OAR volume receiving < 20 Gy.

* Paired-sample T-test.

p = p-value of dosimetric parameters of PG-only and PG+APG in Plan1.

p’ = p-value of dosimetric parameters of PG+APG in Plan1 and PG+APG in Plan2.

**Figure 3:**
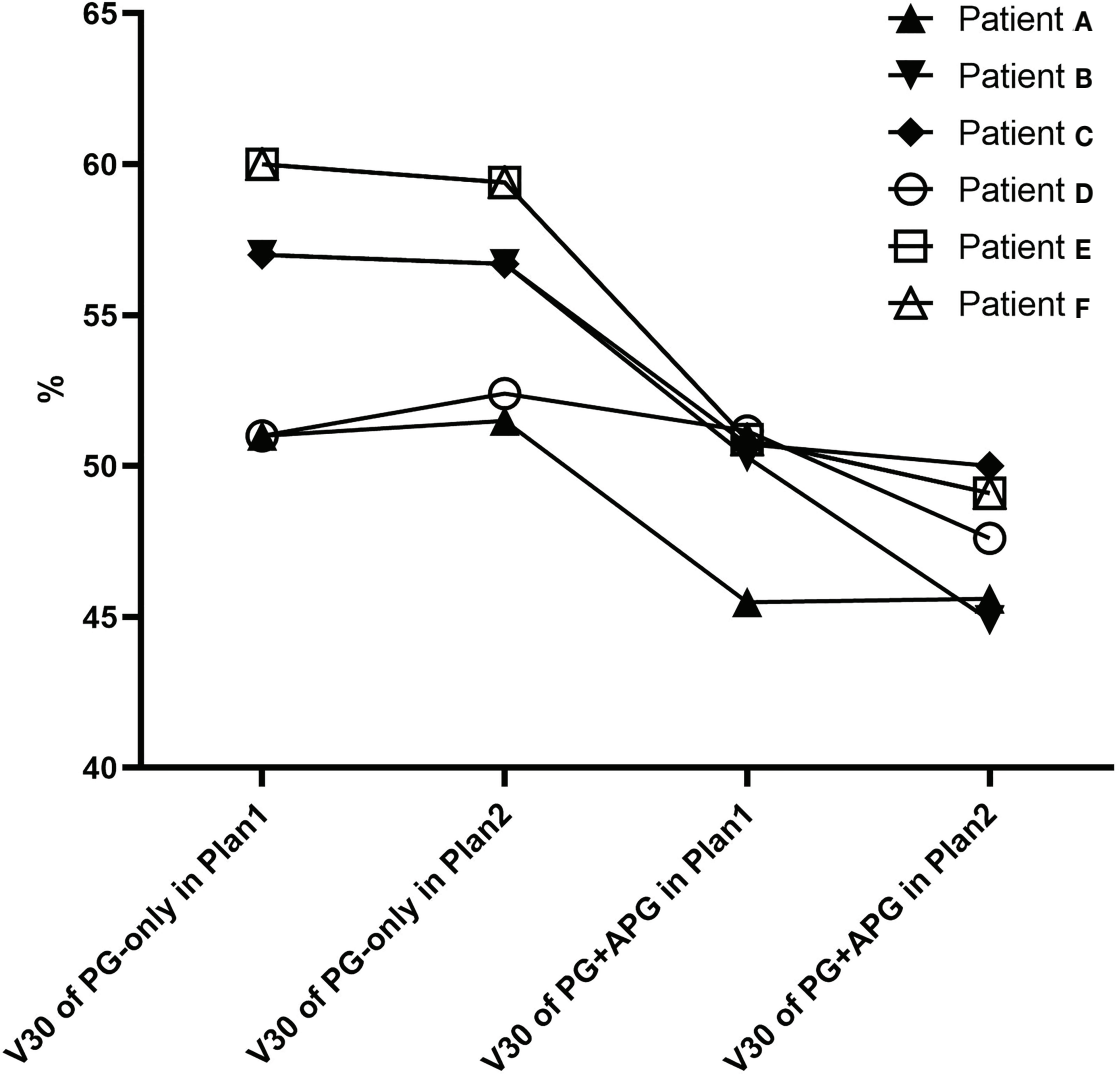
V30 of seven patients that improved and met the PG dose restriction standard in Plan1 (patients E–G) and Plan2.

Overall, more favorable mean dosimetry was observed for the combined structures in Plan1. In Plan1, the PG+APG delineation approach associated with three additional patients who met the dose constraint for V30, resulting in a slight improvement in the rate that met the dose restriction of the PGs (37.5%, 12/32 vs. 46.9%, 15/32; p > 0.05). All three patients had stage III–IVa NPC and grade 0–1 xerostomia, two of whom exhibited bilateral APGs. The maximum diameter of LNs ipsilateral to the APG ranged between 1.2 and 6.9 cm among these patients. In Plan2, the V30 of four additional patients improved to meet the dose criteria, resulting in significant improvement in dose constraint fulfillment rate (37.5%, 12/32 vs. 59.4%, 19/32; p < 0.05). Of these seven patients, V30 of PG-only in Plan1 and that of PG+APG in Plan2 ranged between 51.2% and 61.7% and 41.4% and 49.6%, respectively (**Figure 3**). At 6 months follow-up, five of them exhibited grades 0–1 xerostomia, while two reported grade 2 xerostomia.

### Xerostomia and the predictive value of dosimetric parameters

Grades 0–1 xerostomia was reported in 25 patients (grade 0, n = 9; and grade 1, n = 16), grade 2 xerostomia was reported in 5, while grade 3–4 xerostomia was not observed. Two patients were lost to follow-up. The clinical characteristics of patients based on the development of clinically significant xerostomia are shown in **Table 3**. Patients who underwent induction chemotherapy and concurrent chemoradiotherapy (InC+CRRT) were associated with significantly higher rates of grade 2–3 xerostomia compared to those who underwent CCRT alone (p < 0.05).

**Table 3:** Association between xerostomia and clinical characteristics.

Variable	≥ Grade 2		Grade 0–1		p-value
Sex	n	(%)	n	(%)	0.859
Male	3	60.0%	19	76.0%	
Female	2	40.0%	6	24.0%	
Age					>0.999
≥56	3	60.0%	16	64.0%	
<56	2	40.0%	9	36.0%	
Maximum diameter of unilateral LNs					0.138
>2.5 cm	4	80.0%	9	36.0%	
≤2.5 cm	1	20.0%	16	64.0%	
T-stage					0.364
T1–2	2	40.0%	9	36.0%	
T3–4	3	60.0%	16	64.0%	
N-stage					0.300
N0–1	2	40.0%	7	28.0%	
N2–3	3	60.0%	18	72.0%	
Clinical stage					0.183
I–II	2	40.0%	3	12.0%	
III–IVa	3	60.0%	22	88.0%	
Clinical levels of LN					>0.999
No	0	0.0%	1	4.0%	
II	1	20.0%	7	28.0%	
II–III	4	80.0%	14	56.0%	
II, IV	0	0.0%	2	8.0%	
II–IV	0	0.0%	1	4.0%	
Treatment					0.023
InC+CCRT	3	60.0%	25	100.0%	
CCRT	2	40.0%	0	0.0%	

AUC, area under the curve; 95% CI, 95% confidence interval; PG-only, parotid glands as an organ at risk; PG+APG, the parotid glands and accessory parotid gland as a single organ at risk; Dmean, mean dose; D50, dose of 50% OAR volume; V26, percentage of the OAR volume receiving ≥ 26 Gy; V30, percentage of the OAR volume that received ≥ 30 Gy; V33, percentage of the OAR volume that received ≥ 33 Gy; V45, percentage of the OAR volume that received ≥ 45 Gy.

In Plan1, the AUCs of Dmean, D50, V30, V33, and V20cc of PG+APG tended to increase compared to those of PG-only, although no significant differences were shown. The AUCs of V26 and V45 for PG-only and PG+APG remained similar (**Table 4**).

**Table 4:** Receiver operating characteristic (ROC) analysis of dosimetric parameters in Plan1 for xerostomia.

Variable	AUC/95% CI	p-value
Dmean		0.264
PG-only	0.552 (0.210–0.894)	
PG+APG	0.608 (0.270–0.946)	
D50		0.050
PG-only	0.512 (0.198–0.826)	
PG+APG	0.624 (0.294–0.954)	
V26		>0.999
PG-only	0.616 (0.273–0.959)	
PG+APG	0.616 (0.282–0.950)	
V30		0.596
PG-only	0.600 (0.267–0.933)	
PG+APG	0.640 (0.309 –0.971)	
V33		0.512
PG-only	0.592 (0.265–0.919)	
PG+APG	0.640 (0.306–0.974)	
V45		>0.999
PG-only	0.584(0.263–0.905)	
PG+APG	0.584 (0.252–0.916)	
V20cc		0.952
PG-only	0.472(0.100–0.844)	
PG+APG	0.504(0.161–0.874)	

AUC, Area under the curve; 95% CI, 95% confidence interval; PG-only, parotid glands as an organ at risk; PG+APG, the parotid glands and accessory parotid gland as a single organ at risk; Dmean, mean dose; D50, dose of 50% OAR volume; V26, percentage of the OAR volume receiving ≥ 26 Gy; V30, percentage of the OAR volume that received ≥ 30 Gy; V33, percentage of the OAR volume that received ≥ 33 Gy; V45, percentage of the OAR volume that received ≥ 45 Gy.

## Discussion

The salivary glands consist of three major pairs of glands, namely, the PGs, submandibular glands, and sublingual glands, with 65% of saliva produced by the PGs (17). Given that the submandibular glands are often located within the target volume during RT and that the sublingual glands are usually difficult to recognize, preservation of the PGs is of great importance. The APG functions similarly to the PGs and demonstrates no appreciable histopathological differences (8). To our knowledge, the role of APG preservation during RT for NPC has not been explored. As such, our study represents the first in assessing the effects of treating the APG and PGs as a single OAR in NPC radiotherapy.

Heterogeneity exists in the prevalence and location of the APG in the population, as more than one APG may be found unilaterally, adjacent to a single PG, or deviate from its expected location (18). The prevalence of the APG in our study was 23.53%, which was lower than the 33.8% prevalence found in a recent meta-analysis in Asia (18). In addition, a higher prevalence was observed in male compared to female patients. Unlike the findings of Toh et al. (9), bilateral APGs were observed in the majority of our patients (56.3%). This may be due to the smaller sample size of our study and the higher proportion of male patients.

The mean size of the APG has been reported to be 15.8 × 5.0 mm on CT (19) and can range from the size of a pea to that of a kidney bean, as described in the cadaveric study by Frommer (8). Our results were in accordance with such findings. In the study by Pujol-Olmo et al., the PGs demonstrated a mean height and width of 66.37 and 46.84 mm, respectively (20). A longitudinal volumetric study found that the mean volume of PGs ranged between 28.7 and 32.2 ml (21), which was consistent with our data (mean, 29.4 ± 1.3 ml; range, 15.8–47.7 ml). While the PGs were found to be larger compared to the APG, there was a great extent of overlap with PTV54Gy (mean, 28.36%), rendering most of the organ exposed to a radiation dose ≥54 Gy. In contrast, no overlap between the APG and the target volume was observed, indicating that protection of the APG may be easily achieved. A study revealed that partial volume thresholds for the prediction of reduced salivary flow were V45 < 24% and V30 < 45% (22). In Plan1 of our study, V45 and V30 were 44.16% and 62.00%, respectively, to the PGs only, which reduced to 39.18% and 57.41%, respectively, upon involvement of the APG. When the APG was intentionally protected in Plan2, improvements were observed in all dosimetric parameters to the organ, besides V26, which remained higher than the threshold. This may be explained by the large proportion of patients with locally advanced NPC in our study and the relative proximity in location of PG+APG to the target volume.

Dmean, V30, and D50 represent the most critical dosimetric predictors of parotid function impairment in NPC radiotherapy (11). We found that the PG+APG delineation approach resulted in a significant improvement in the aforementioned parameters in both Plan1 and Plan2 (p < 0.001). In Plan1, the reductions in the mean value of Dmean and D50 were 1.84 and 3.96 Gy, respectively, while that of V30 was approximately 5%. Among the patients who did not meet the dose constraint, only three patients improved and met the criteria by the addition of APG in Plan1. This may be due to 1) the close location of PGs to the treatment area, resulting in a relatively high volume overlap with PTV54Gy, and 2) a large percentage of level II lymph node involvement. When the APG was outlined and protected in Plan2, the rate that met the dose constraints of the PGs significantly improved from 37.5% to 59.4% (p < 0.05), and the mean decreases in Dmean, D50, and V30 were 3.1 Gy, 5 Gy, and approximately 6.5%, respectively, which were greater than those observed in Plan1.

After IMRT, saliva flowrate often significantly decreases in NPC patients (0.10 ml/min vs. 0.57 ml/min at baseline) and only partially recovers a year later (23). Based on the study by Poon et al. (24), approximately 20% of NPC patients developed chronic grade 2–3 grading xerostomia following IMRT, which is consistent with our results (5/30, 16.67%). Grade 3 xerostomia was not observed in our patients. Han et al. demonstrated the difference in the influence of spatial dose patterns on the salivary glands on xerostomia development and recovery, with recovery showing increased importance towards subvolumes that received lower radiation doses (25). Without deliberate protection of the APG, we found that the radiation exposure of the APG ranged between 20 and 30 Gy (Dmean, 24.79 ± 0.85 Gy). By contouring the APG as an OAR, this reduced to 10–20 Gy (Dmean, 14.22 ± 0.41 Gy). As such, our findings proposed that APG sparing during RT may facilitate better recovery from xerostomia.

In terms of the predictive factors of xerostomia, Gabrys et al. found that Dmean of the PGs failed to recognize patients at risk of grade ≥ 2 xerostomia (26), and no dosimetric parameters (including Dmean, V20, V30, V40, V50, and V60) were reported to significantly associate with xerostomia in the studies by Sommat et al. (27) Our study corroborated with such results and found no significant associations between xerostomia and any of the dose–volume parameters of PG+APG (AUC < 0.700). However, we observed that the AUCs of nearly all dosimetric parameters, especially D50 (p = 0.050), of the PG+APG delineation approach tended to improve compared to that of the PG-only delineation approach. Furthermore, a recent study found V25, V30, V35, V45, and Dmean to the PGs as independent predictive factors for xerostomia, although with low assessment ability (AUC < 0.700) (28). Our results may be mainly attributed to the more accurate reflection of the salivary gland volume with the addition of the APG. In Plan2, the dosimetry of seven patients improved to meet the dose restriction standards of the PGs, and five of them exhibited 0–1 grade xerostomia, indicating that inclusion of the APG resulted in increased association between PG+APG dosimetry and xerostomia severity. Given the lack of prospective analyses, the effects of adding APG as an OAR on the prediction of xerostomia require further evaluation. Nonetheless, it is well known that the incidence of xerostomia can be influenced by a multitude of factors, including clinical features and treatment strategies (29). We also found that patients who underwent InC+CRRT were more likely to develop chronic xerostomia. Dosimetric data alone may thus be insufficient for the accurate prediction of late xerostomia. While considerable research effort has been put into this subject (30, 31), further studies involving clinical, molecular, and radiological variables are warranted.

## Conclusions

Our study presented a novel approach to PG delineation during RT for NPC by considering the inclusion of the APG. Our results showed that consideration of the APG as a homologous part of the PG resulted in a significant improvement in the dosimetry of the PGs, particularly when the APGs were intentionally protected during RT. None of the dosimetric parameters were predictive for xerostomia; however, the AUCs of the majority of the parameters tended to increase with the PG+APG delineation approach. Our findings thereby suggest the benefits of considering the APG and PGs as a single OAR during RT for NPC and demonstrate its potential to better reflect the long-term outcomes of such patients. Due to the potential biases of a retrospective study, further prospective research is needed to verify our findings.

## Data availability statement

The raw data supporting the conclusions of this article will be made available by the authors, without undue reservation.

## Ethics statement

The studies involving human participants were reviewed and approved by Sun Yat-sen Memorial Hospital Ethics Committee. The ethics committee waived the requirement of written informed consent for participation.

## Author contributions

X-LY and JH: conceptualization and methodology development. X-LC: data analysis and original draft preparation. X-LY, JH, J-TS, and J-SC: data acquisition. X-LY: draft editing. Y-ML and S-MB: methodology supervision and draft revising. All authors contributed to the article and approved the submitted version.

## Conflict of interest

The authors declare that the research was conducted in the absence of any commercial or financial relationships that could be construed as a potential conflict of interest.

## Publisher’s note

All claims expressed in this article are solely those of the authors and do not necessarily represent those of their affiliated organizations, or those of the publisher, the editors and the reviewers. Any product that may be evaluated in this article, or claim that may be made by its manufacturer, is not guaranteed or endorsed by the publisher.
